# High Thermal Performance Ultraviolet (368 nm) AlGaN-Based Flip-Chip LEDs with an Optimized Structure

**DOI:** 10.3390/nano14030267

**Published:** 2024-01-26

**Authors:** Guanlang Sun, Taige Dong, Aixin Luo, Jiachen Yang, Ying Dong, Guangda Du, Zekai Hong, Chuyu Qin, Bingfeng Fan

**Affiliations:** 1 School of Physics and Optoelectronic Engineering, Foshan University, Foshan 528225, China2112205037@stu.fosu.edu.cn (Y.D.);; 2 Guangdong-Hong Kong-Macao Joint Laboratory for Intelligent Micro-Optoelectronic Technology, Foshan University, Foshan 528225, China

**Keywords:** GaN, indium tin oxide (ITO)/Al reflective mirror, 368 nm

## Abstract

In this study, we have fabricated a 368 nm LED with an epitaxial Indium Tin Oxide (ITO) contact layer. We analyze the thermal performance of the flip-chip LED with a symmetric electrode and metal reflective layer, applying ANSYS to build a coupled electro-thermal finite element model (FEM) of the temperature distribution in the multiple quantum wells (MQWs). We compare our system with the traditional Au-bump flip-chip LED and a flip-chip LED with a Distributed Bragg Reflector (DBR) layer. The simulation results have shown that the flip-chip LED with a metal reflective layer and symmetric electrode exhibits better heat dissipation performance, particularly at high input power. The influence of the insulating layer on the LED chip junction temperature is also examined. The simulation data establish an effect due to the thermal conductivity of the insulating layer in terms of heat dissipation, but this effect is negligible at an insulation layer thickness ≤1 µm.

## 1. Introduction

In recent years, UV light-emitting diodes (LEDs) have gained significant attention because of their wide ranging potential applications [1,2,3,4,5]. Most of the applications of UV LEDs, such as UV curing, require a high output power, and the size of the UV LED chip is consequently designed to be larger in order to operate at a higher input power [6,7,8]. An increase in input power is accompanied by appreciable heat generation as ca. 80% of the LED chip input power is transferred as heat. In the case of UV LEDs, this value is even higher [9,10,11]. The junction temperature of the LED chip affects optical and electrical performance, resulting in a drift of the wavelength which impacts on the reliability and productive life of the LED chip as key factors that determine ultimate application [12,13,14,15]. In improving the output power of the LED chip, it is essential that the thermal properties are enhanced to deliver optimum performance. The flip-chip LED structure was proposed to address issues associated with optical output power and thermal performance [16,17,18,19]. The light associated with the flip-chip LED is extracted from the transparent sapphire side, without an electrode barrier, and the total reflection is reduced due to the refractive index of sapphire, resulting in greater light efficiency. The heat generated by the flip-chip LED is directly dissipated from the GaN layer to the submount through the solder, without passing through the thick sapphire substrate, which exhibits a low thermal conductivity [20,21,22,23].

Previous research on thermal performance has mainly focused on the traditional Au bump flip-chip LED [24,25,26,27]. An increase in the number of Au bumps can improve heat dissipation as the contact area between the LED chip and the submount is increased. However, increasing the number of Au bumps introduces processing difficulties, and may also increase the reverse leakage current. This has limited possible improvements in the thermal performance of Au bump flip-chip LEDs. In order to address these limitations, the use of a symmetric electrode was proposed, which when combined with the submount, serves to appreciably improve thermal performance. This LED arrangement requires the introduction of an insulation layer, but the resultant impact on the thermal performance of the LED chip is unclear.

In this study, we have fabricated a 368 nm LED with an epitaxial Indium Tin Oxide (ITO) contact layer, and have analyzed the thermal performance of this flip-chip LED with a symmetric electrode. The ITO transparent electrode has excellent transparency and low resistivity, and it is widely used in LED chips. The use of ITO thin film research has been proven widely to achieve LED photoelectric conversion efficiency and improve optical power output [28,29,30].We have constructed a 3D finite element model for this LED chip arrangement, analyzing thermal performance which is compared with the traditional Au-bump flip-chip LED. The influence of an insulating layer on the LED chip junction temperature has also been addressed.

## 2. Materials and Methods

### 2.1. Fabrication of the 368 nm Flip-Chip LED

**Figure 1:**
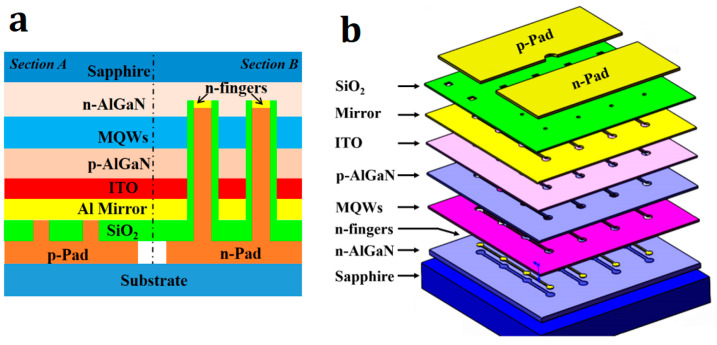
Structure of the flip-chip LED with metal reflective layer and symmetric electrode: (**a**) cross-sectional view; (**b**) schematic of the flip-chip LED with symmetric electrode.

Prior to fabricating the 368 nm flip-chip LED, an epitaxial ITO contact layer was deposited on the LED epitaxial layer, forming good ohmic contact with p-GaN. The ITO thin films were grown on c-plane sapphire substrates by metal organic chemical vapor deposition (MOCVD) using a Veeco Emcore400 system. Trimethyl Indium (TMIn) and Tetrakis-Dimethylamino Tin (TDMASn) were employed as the precursors of In and Sn; O_2_ and Ar served as carrier gases. The samples were grown at ca. 500 °C with a thickness of ca. 50 nm [31,32]. The structure of the 368 nm flip-chip LED with a metal reflective layer and a symmetric electrode is shown in Figure 1. First, the ITO layer was partially removed using a HCl and HNO_3_ solution (10:1), followed by inductively coupled plasma (ICP) etching to form the mesa structures with an etch depth of 1.35 μm in order to expose the n-GaN surface. Then, the metal “n-fingers” were deposited on the n-GaN surface to extend the current, and the Cr/Al/Ni/Au (2/2500/200/600A) metal reflective layer was deposited on the ITO surface to form good ohmic contact and a UV high reflective interface. The insulation layer (1 μm-thick SiO_2_) was deposited on this structure using plasma-enhanced chemical vapor deposition. The SiO_2_ on the “tips” of the n-fingers and the reflective layer was partially removed by a 7:1 buffered oxide agent. The symmetric electrode was then deposited and was connected to the n-fingers and the reflective layer through the holes in the insulating layer. The symmetric electrode was thickened by electroplating 1 μm Au to strengthen the electrical connection and enable heat conduction. Finally, the chip was soldered to a ceramic submount by Au/Sn. The simulation parameters are shown in Table 1.

**Figure 2:**
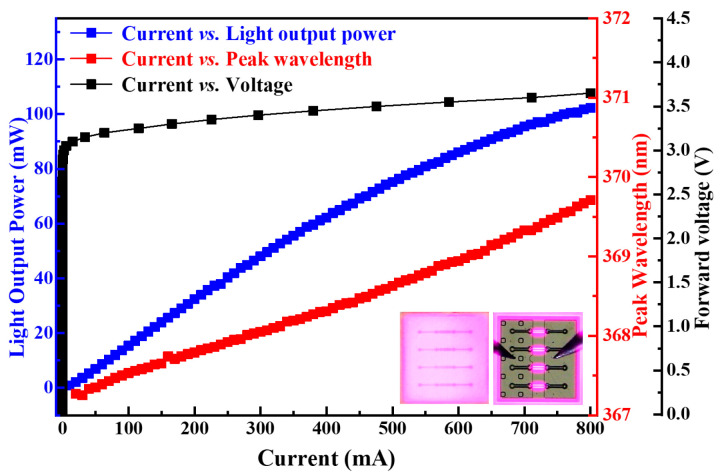
Voltage, light output power and wavelength peak of the ITO contact layer and flip-chip light emitting diode with symmetric electrode for different input currents.

The electrical and optical characteristics of the flip-chip LED without packaging was measured using an IPT 6000-LED Probing System with an Integrated Tester. The voltage, LOP (light output power) and WP (wavelength peak) of the flip-chip UV-LED for different input currents are presented in Figure 2. The image insert shows the bare LED chip. It can be seen that the forward voltage of the LED chip is 3.43 V at a 350 mA operating current. As the current increases, the output power of the flip-chip UV-LED shows an initial near linear increase and a gradual lower dependence as the input current reaches 700 mA. The wavelength peak of the flip-chip UV-LED is 368.19 nm at 350 mA, shifting to 369.71 nm at 700 mA. The observed response demonstrates that the emitting wavelength of the fabricated flip-chip UV-LED is relatively stable.

### 2.2. FEM Model and Parameters

In our thermal analysis, we built a coupled electro-thermal 3D finite element model of the flip-chip LED according to the chip structure illustrated in Figure 1 [33,34,35,36]. For the purposes of comparison, a thermal simulation of the traditional Au-bump flip-chip LED and a flip-chip LED with a Distributed Bragg Reflector (DBR) layer and symmetrical electrode were considered. In related research, it was shown that the heat generated by the LED chip is mainly due to Joule heat associated with the high resistivity p-GaN and the non-radiative recombination of multiple quantum wells (MQWs) [37,38,39]. The distribution of Joule heat and non-radiative recombination is closely related to the current density distribution. In order to ensure that the thermal simulation is reliable, we have employed the electro-thermal coupling mode and input operating current as the heat source for the chip. Moreover, in order to facilitate processing, the Joule heat is taken as the heat source in our experiment, and the corresponding resistivity was set according to the conversion efficiency. In our simulation experiment, the temperature of the bottom surface of the ceramic submount was fixed at 50 °C, where the other surface of the chip was adiabatic. Only heat conduction was considered, ignoring thermal convection and thermal radiation.

**Figure 3:**
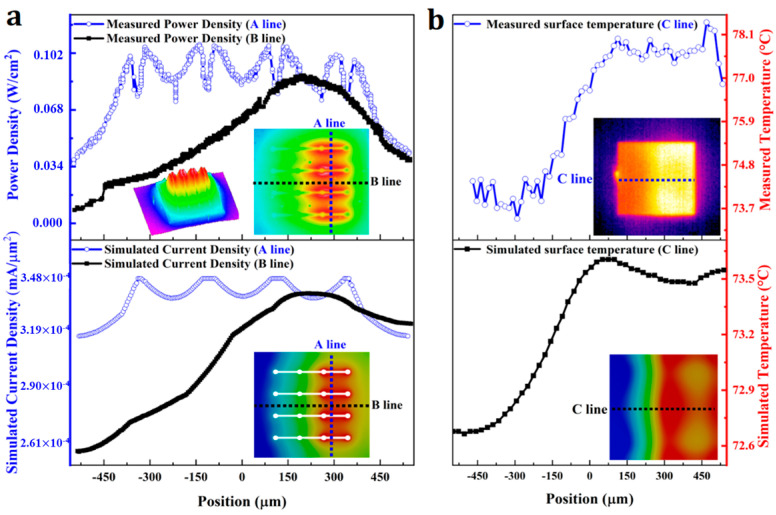
ITO contact layer and flip-chip light emitting diode with symmetric electrode: (**a**) The optical output power curve obtained by experimental measurement and the current density curve obtained from simulation; (**b**) The chip surface temperature curve obtained by experimental measurement and from simulation.

In order to verify rationality of the established finite element model, we utilized a flip-chip with a metal reflective layer as a test case, and conducted experimental measurements of optical power density and surface temperature. The results are presented in Figure 3. It can be seen that the optical power density curve measured by the experiment and the current density curve obtained from the simulation exhibit essentially the same trend, which also applies to the measured and simulated chip surface temperature response. The experimental results and the simulation results are in good agreement, and the finite element model can be deemed reasonable and effective.

## 3. Results and Discussion

**Figure 4:**
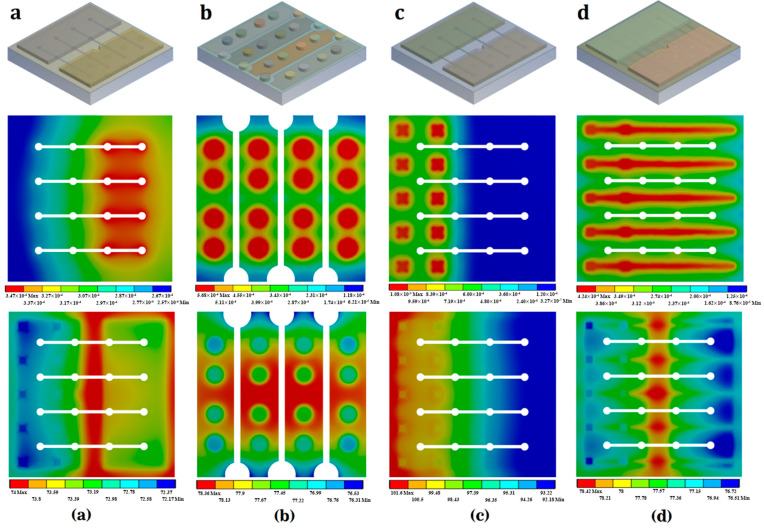
Current density/temperature distribution in the MQWs layer (at 350 mA): (**a**) Flip-chip with a metal reflective layer; (**b**) Traditional Au-bump flip-chip; (**c**) Flip-chip with DBR reflective layer (without p-fingers); (**d**) Flip-chip with DBR reflective layer (with p-fingers).

The simulated current density/temperature distribution in the MQWs layer of the flip-chip LED shown in Figure 4 refers to an input current of 350 mA. The top row of images illustrate the 3D finite element models for the four flip-chip LED arrangements. The current density and temperature distributions are presented in the middle and bottom rows, respectively. Through a comparison of the current density and temperature distribution profiles, it is obvious that the temperature distribution is closely related to the current density distribution. The temperature distribution on the surface of the chip in Figure 4a reveals a concentration of high temperature in the middle section, with a low value at the edge area. The chip temperature is clearly affected by the symmetric electrode which is soldered to the submount and serves as the main channel for heat dissipation. The temperature is accordingly lower in the symmetric electrode area. The temperature is much lower in the area near the insulating layer holes, through which the heat is directly conducted to the ceramic submount. It can be concluded that the symmetric electrode and the insulating layer have a significant effect on heat dispersion. The current distribution shown in Figure 4a is the most uniform. Therefore, the structural design of “model a” provides the most effective heat dissipation, when compared with the other three models.

**Figure 5:**
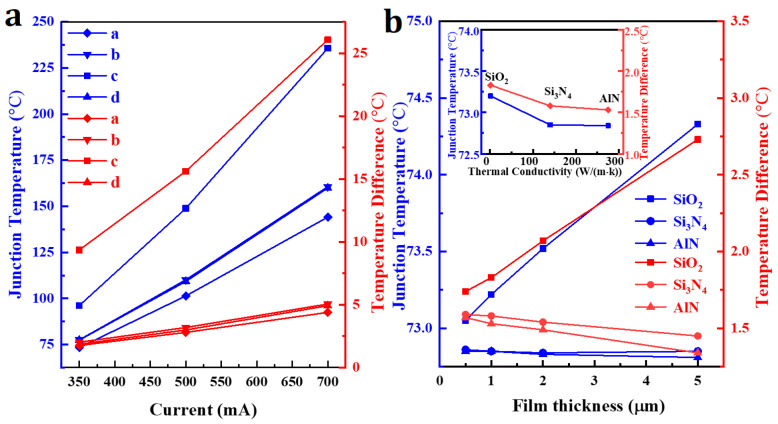
(**a**) Junction temperature and temperature differential in the LED chip at different input current. (**b**) The junction temperature and temperature differential of the LED chip with variations in the thickness of three insulating layers (SiO_2_, Si_3_N_4_, and AlN).

The junction temperature and temperature differential associated with the LED chip is presented as a function of input current in Figure 5a. An increase in the input current is accompanied by an increase in both the junction temperature and the temperature differential. The values of both parameters are lowest for “model a”, indicating that the flip-chip LED with a metal reflective layer and symmetric electrode offers the best heat dissipation across the range of input power (350–700 mA).

In order to assess the influence of the insulating layer on heat dissipation in the case of the flip-chip LED with a metal reflective layer and symmetric electrode, we altered both the thickness and thermal conductivity of the insulating layer and measured the junction temperature and temperature differential. As shown in Figure 5b, increasing the thickness of the SiO_2_ layer, with a low thermal conductivity, resulted in an increased junction temperature and differential. Silica layer thickness is accordingly an important factor that reduces the heat dissipation capability of the chip. The use of Si_3_N_4_ or AlN as insulating layers with a higher thermal conductivity resulted in less variation of temperature with layer thickness, and the temperature differential showed a slight decrease. The results demonstrate that when the thermal conductivity of the insulating layer is relatively high, the thickness of the insulating layer has little influence on the thermal performance of the flip-chip LED. Generally, the thickness of the insulating layer in current flip-chip LEDs is ca. 1 μm. At this thickness, the effect of thermal conductivity of the insulating layer on junction temperature and temperature differential is relatively small, as shown in Figure 5b.

## 4. Conclusions

In this study, we have evaluated the thermal performance of a new type of ITO contact layer and flip-chip LED with symmetric electrodes. At an input current of 350 mA, the junction temperature and temperature differential of this flip-chip LED arrangement are smaller than the traditional Au-bump flip-chip LED and DBR reflective layer flip-chip LED. Moreover, the current distribution in the MQWs layer is established as the most uniform. Therefore, this structural design better facilitates heat dissipation than the other three options. Such versatile strategy has universal applicability and can be applied to other types of flip-chip LEDs. At high input power, flip-chip LEDs with metal reflective layers and symmetric electrodes deliver better heat dissipation performance. We also examined the influence of the insulation layer on the LED chip junction temperature. Although the thermal conductivity of the insulation layer (SiO_2_, Si_3_N_4_, and AlN) can have an appreciable effect on heat dissipation performance, at ca. 1 μm layer thickness, the effect is minimal. The results of this study can provide guidance for the further development of LEDs with enhanced heat dissipation performance.

## Figures and Tables

**Table 1:** Parameters used in the simulations.

Material/Layer	Thickness/µm	Thermal Conductivity (W/(m·k))	Resistivity/(ohm·m)
Sapphire	150	46	-
n-GaN	2.7	130	4 × 10^−6^
MQWs	0.1	130	0.05
p-GaN	0.12	130	20
ITO	0.1	11	8 × 10^−6^
Mirror	0.35	-	-
n-fingers	0.35	-	-
SiO_2_	1	1.4	-
PAD	1.5	-	-

## Data Availability

Data are contained within the article.
